# The Economic and Humanistic Burden of Pediatric-Onset Multiple Sclerosis

**DOI:** 10.36469/001c.37992

**Published:** 2022-10-18

**Authors:** Nupur Greene, Lita Araujo, Cynthia Campos, Hannah Dalglish, Sarah Gibbs, Irina Yermilov

**Affiliations:** 1 Health Economics & Value Assessment, Sanofi, Cambridge, Massachusetts; 2 PHAR (Partnership for Health Analytic Research), LLC, Beverly Hills, California

**Keywords:** pediatric multiple sclerosis, quality of life, healthcare resource utilization, epidemiology

## Abstract

**Background:** Multiple sclerosis (MS) is a chronic inflammatory autoimmune disease of the central nervous system. Pediatric-onset MS (POMS), defined as onset of MS before 18 years of age, is estimated to account for 2% to 5% of the MS population worldwide. **Objectives:** To conduct a literature review focused on the healthcare resource utilization and cost as well as quality-of-life (QOL) outcomes among patients with POMS. **Methods:** We conducted a systematic literature review of English-language studies published after September 2010 in MEDLINE and Embase to describe the global economic healthcare resource utilization and costs and humanistic (QOL) burden in patients with POMS. **Results:** We found 11 studies that reported on healthcare resource utilization, cost, or insurance coverage and 36 studies that reported on QOL outcomes in patients with POMS. Patients with POMS had higher rates of primary care visits (1.41 [1.29-1.54]), hospital visits (10.74 [8.95-12.90]), and admissions (rate ratio, 4.27 [2.92-6.25];OR, 15.2 [12.0-19.1]) compared with healthy controls. Mean per-patient costs in the United States were 5907acrossallsettingsperyearoffollow−upbetween2002and2012;meancostsperhospitalstaywere38 543 (in 2015 USD) between 2004 and 2013. Three studies reported psychosocial scores between 71.59 and 79.7, and 8 studies reported physical health scores between 74.62 to 82.75 using the Pediatric Quality of Life Measurement Model (PedsQLTM). Twelve studies used the PedsQL™ Multidimensional Fatigue Scale. Mean scores on the self-reported general fatigue scale ranged from 63.15 to 78.5. Quality-of-life scores were lower than those of healthy controls. **Discussion:** Our review presents a uniquely broad and recent overview of the global economic and humanistic burden of patients with POMS. Additional research on healthcare resource utilization and cost would provide a more robust understanding of the economic burden in this population. **Conclusions:** Healthcare resource utilization and costs are high in this population, and patients report reduced QOL and significant fatigue compared with healthy children and adolescents.

## BACKGROUND

Multiple sclerosis (MS) is a chronic inflammatory autoimmune disease of the central nervous system with onset of symptoms usually occurring in adults between 20 to 40 years old.^1,2^ Pediatric-onset MS (POMS), defined as onset of MS before 18 years of age, is estimated to account for 2% to 5% of the MS population worldwide.^1,3^ Incidence and prevalence vary by geography and patient age. A recent systematic literature review and meta-analysis reported that incidence ranges from 0.05 to 2.85 per 100 000 children across 12 countries (in North America, Europe, Middle East, and Asia); pooled global incidence was reported to be 0.87 per 100 000 children.^4^ Prevalence ranged from 0.69 to 26.92 (pooled: 8.11) per 100 000 children.^4^ Incidence and prevalence are higher in the United Arab Emirates, where incidence was reported to be 2.30 per 100 000 children between 10 to 14 years old and 7.20 per 100 000 adolescents between 15 to 19 years old, and prevalence was reported to be 30.7 per 100 000 children.^5^

Children have higher relapse rates and a greater burden of lesions detected by magnetic resonance imaging than adults,^6,7^ but disease progression tends to be slower. Due in part to the younger age at disease onset, children reach disability milestones and secondary progression earlier than adults. Studies have also shown patients with POMS report reduced health-related quality of life (QOL) compared with healthy controls.^8^ Similar to adults, children with POMS may also experience significant fatigue (some estimates report 20%-75% of patients),^9^ resulting in further psychological challenges and reduced QOL.^10^

Recent reviews have focused on characterizing how the disease course differs from adult-onset MS^1,11,12^ and guiding physicians on disease management.^1^ Reviews on healthcare costs of adult patients with MS have also been conducted in Spain^13^ and the United States,^14^ but no global reviews have explored the economic burden (eg, healthcare resource utilization and costs) in patients with POMS. Studies on QOL outcomes can provide a clear description of how MS impacts emotional, social, and physical functioning of pediatric patients. Reviews of QOL studies in POMS have focused on fatigue,^10^ comparing neuropsychological correlates of adults vs children with MS,^8^ and cognitive function.^9,15^

This literature review focuses on the healthcare resource utilization and cost as well as QOL outcomes among patients with POMS. Reporting on these outcomes together can provide a comprehensive overview of the societal and individual costs of the disease, including the decrement in QOL.

## METHODS

As part of a larger study, we conducted a systematic literature review to identify published information on the epidemiology, treatment patterns, and clinical, humanistic, and economic disease burden of 10- to 17-year-old patients with POMS. We searched MEDLINE (via PubMed) and Embase on September 27, 2020, to identify studies written in English, published in the last 10 years (September 27, 2010–September 27, 2020), that presented data on human patients with POMS between 10 to 17 years old from any country, and included at least 1 outcome of interest (epidemiology, treatment patterns, morbidity, mortality, comorbidities, QOL, healthcare resource utilization, healthcare costs, and health insurance coverage). We present studies that reported data on healthcare resource utilization, healthcare costs, health insurance coverage, and QOL to present all societal and individual costs (economic and humanistic) of POMS.

MeSH and text words associated with POMS (eg, “pediatric onset multiple sclerosis”[tw], “POMS”[tiab], “pediatric multiple sclerosis”[tw]), children (eg, “child”[Mesh], “adolescent”[Mesh], kid[tw]), economic disease burden (eg, “cost of illness”[Mesh], “economic burden of disease”[tw], “healthcare utilization”[tw], “healthcare cost*”[tw]), and QOL (eg, quality of life”[Mesh], “HRQOL”[tw], “health related quality of life”[tw]) were used. Full search strings are presented in **Supplemental Table S1.**

Researchers experienced in literature reviews screened articles in 2 phases: an initial title and abstract screen followed by a full-text screen. Articles that stratified data by the pediatric population and were not conference abstracts or pharmacokinetics/pharmacodynamics, case studies, or in vitro studies were included. References of these included articles and relevant literature reviews on POMS were mined (ie, reviewed and screened) to ensure no key articles were missed. We conducted the review using DistillerSR (Evidence Partners, Ottawa, Canada), a systematic review program. From all studies that met the inclusion criteria, we abstracted the study design, patient and control group population (including age ranges and MS type if specified), data source, country, dates of data collection, and relevant data on each outcome of interest. **Supplemental Figure S1** depicts the number of articles screened and included.

## RESULTS

Our full search resulted in 4599 unique articles, of which 202 were included in the review. Eleven studies reported on economic burden, including healthcare resource utilization and cost and/or health insurance coverage (**Supplemental Table S2**) and 36 studies reported on QOL outcomes (**Supplemental Table S3**), which are described in this manuscript. Some studies reported on more than 1 of these outcomes. Key findings across all studies are highlighted in **Table 1.**

**Table 1.** Summary of Key Findings for Economic and Humanistic Outcomes Compared With Healthy Controls

**POMS Patient Outcomes Reported by ≥2 Studies**	**POMS Patient Outcomes Compared With Healthy Controls**
Select economic outcomes	
Inpatient admissions/hospitalizations	
1.2-6.0 mean hospital admissions per patient over a 10-year period^16,17^	Admissions RR 4.27 (2.92-6.25)^18^ Admissions OR 15.2 (12.0-19.1)^19^ 16.5% vs 2.0% hospitalized^19^ 19.4% vs 0.0% hospitalized^20^
Other utilization	
7-13 physician visits over an unspecified follow-up period^20,21^	Primary care visits RR 1.41 (1.29-1.54), hospital visits RR 10.74 (8.95-12.90)^18^
Healthcare costs^a^	
58%-67% private insurance^22,23^	NR
Select humanistic outcomes	
Quality of life^b^	
63.35-72.04 mean score on emotional component of PedsQL^TM^ 4.0^24–27^ 58.15-66.88 mean score on school component of PedsQL^TM^ 4.0^24,26,28^ 79.60-88.73 mean score on social component of PedsQL^TM^ 4.0^24–27^	74.62-82.75 vs 89.90 mean score on physical health component of PedsQL^TM^ 4.0^20,24–30^ 71.59-79.70 vs 79.57 mean score on psychosocial summary score of PedsQL^TM^ 4.0^20,24–30^
Fatigue	
23%-61.1% self-reported fatigue^31,32^	63.15 vs 74.20-77.64 mean score on self-reported general fatigue scale^27,28,33–35^ 19.8% vs 2.9% self-reported severe fatigue^27^ 27.1-30.6 mean FSS^c^ vs 21.5 median FSS^36–38^ 43.8% vs 0.0% reported chronic fatigue^39^ 32.52 vs 23.10 mean scores on the Modified Fatigue Impact Scale^40^
Other^d^	
NR	43.4 vs 67.5 mean exercise min/wk on GLTEQ^41^ 36.00 vs 65.00 median exercise min/wk on GLTEQ^42^

Abbreviations: FSS, Fatigue Severity Scale; GLTEQ, Godin Leisure Time Exercise Questionnaire, NR, not reported; OR, odds ratio; RR, rate ratio.

The left column reports outcomes when ≥2 studies presented that outcome (allowing us to present a range). The right column presents outcomes compared with healthy controls. Definition of healthy controls varied by study (eg, healthy matched controls, age- and sex-matched healthy controls).

^a^Other than patient insurance type, no 2 studies reported on the same healthcare costs and therefore no ranges can be presented. No studies compared costs with healthy controls.

^b^Overall, higher mean scores (out of 100) indicate better health-related QOL, although no standard categories exist^27^ Outside the MS literature, Beverung et al^34^ classified a score of 81 to 100 as “better quality of life” and below 60 as “impaired quality of life.”

^c^No standard categories exist for the FSS. Higher scores represent greater fatigue severity.

^d^No 2 studies reported on the same other humanistic outcomes and therefore no ranges can be presented.

### Economic Burden

Eleven studies presented data on healthcare resource utilization, healthcare cost, and/or health insurance coverage (**Supplemental Table S2**). Studies most often collected data from patient populations in the United States (n = 4), followed by Canada (n = 2), Denmark (n = 2), Brazil (n = 1), and Switzerland (n = 1); 1 study collected data from multiple countries (United States, Italy, Russia, Argentina, France, Canada, Tunisia, and Venezuela). Seven studies included data only on patients at least 18 years old (diagnosed with MS as children), and 2 studies included patients who were slightly older (≤19 years) or slightly younger (≤17 years). The remaining 2 studies included patients of any age but stratified data by patients 18 years old or younger. Five studies included control groups, most commonly age- and sex-matched healthy controls. Outcomes from each study are presented in **Tables 2 and 3**.

**Table 2.** Healthcare Resource Utilization Study Outcomes

**Author (Year)**	**Country**	**Healthcare Resource Utilization**
Marrie et al (2020)^20^	Canada	Mean (SD) duration of follow-up (y) Study period: 6.16 (3.96) For HRQoL assessments: 2.6 (2.04) Median (IQR) number of HRQoL assessments (patients with MS <18 y vs healthy controls): 3 (2-4) vs 1 (1-1) 19.4% of patients with MS (vs 0 healthy controls) hospitalized between first and last HRQoL date (total 16 times) Median (IQR) number physician visits from first to last HRQoL date (patients with MS vs healthy controls): 13 (6-27) vs 0 (0.0) Rate of ambulatory visits rate ratio (95% CI) vs healthy controls: 1.49 (0.99-2.25)
Marrie (2019)^19^	Canada	Baseline utilization among prevalent cases (in the year before index date, patients with MS ≤18 y vs age-, sex-, and region-matched healthy controls) Hospitalized: 16.5% vs 2.0% Median (IQR) number of physician visits: 9 (5-16) vs 3 (1-6) Crude annual rate (95% CI) of utilization per 100 person-y (patients with MS vs age-, sex-, and region-matched healthy controls) Hospitalizations: 34.1 (25.5-44.8) vs 3.1 (2.0-4.6) Ambulatory physician visits for MS cohort ranged from 828.2 (783.9-874.2) to 1703.3 (1643.9-1764.4), ≥3-fold higher in the MS cohort than in matched cohort Adjusted analysis (MS vs healthy controls) Odds ratio (95% CI) of any hospitalization: 15.2 (12.0-19.1) Rate ratio (95% CI) of ambulatory physician visits: 4.58 (4.26-4.92) Healthcare utilization among incident cases (from time of diagnosis) also included
Boesen et al (2020)^18^	Denmark	Mean utilization per year for patients with MS <18 y Primary care visits: 4.6 Hospital visits: 5.9 Hospital admissions: 0.5 Rate ratio (95% CI) for healthcare utilization at 1-y follow-up for patients with MS vs age- and sex-matched healthy controls Primary care visit: 1.41 (1.29-1.54) All hospital visits: 10.74 (8.95-12.90) Hospital admissions only: 4.27 (2.92-6.25) 30-day and 5-y follow-up periods, and MS vs non–brain-related chronic disease rate ratios also included
Boesen (2019)^43^	Denmark	Mean, median (SD, range) utilization for patients with MS <18 y vs age- and sex-matched controls No. of hospital infections: 0.0055, 0 (0.074, 0-1) vs 0.0077, 0 (0.099, 0-2) Antibiotic prescriptions: 0.73, 0 (1.2, 0-9) vs 0.59, 0 (1.01, 0-6) Tests in primary care: 0.94, 0 (1.47, 0-8) vs 0.79, 0 (1.27, 0-9) All exposures combined: 1.45, 0 (1.90, 0-11) vs 1.19, 0 (1.54, 0-10) HR for infections for MS children and controls also included
von Wyl et al (2020)^21^	Switzerland	Median (IQR) number of visits during follow-up among patients with MS <18 y: 7 (4-10) (median [IQR] y follow-up: 6 [3.1-10.1])
Lavery et al (2016)^16^	US	Mean, median (SD, range) utilization per patient with MS <19 y over the study period Hospital admissions: 6.0, 4.0 (6.1, 1-8) Length of stay per admission, days: 5.5, 4.0 (7.8, 4-4) Mean annual rate of hospital admission per 10 000 2004: 3.47 2013: 5.32
Wright et al (2017)^17^	US	Mean (range) utilization over study period among patients with MS <18 y Total visits: 26.1 (1-308) Outpatient visits: 22.7 (0-294) Inpatient stays: 1.2 (0-6) Emergency visits: 2.1 (0-40) MRIs: 5.6 (1-32)
Krupp (2016)^44^	US, Italy, Russia, Argentina, France, Canada, Tunisia, Venezuela	Hospitalized for initial relapse (% US, ROW) among patients with MS Preadolescents (<12 y): 86.7, 77.1 Adolescents (12-17 y): 46.6, 58.3

Abbreviations: CI, confidence interval; HR, hazard ratio; HRQoL, health-related quality of life; IQR, interquartile range; MRIs, magnetic resonance imaging; ROW, rest of world.

Control group data reported as applicable to outcome of interest.

**Table 3.** Healthcare Cost and Insurance Study Outcomes

**Author (Year)**	**Country**	**Healthcare Costs**
Maia Diniz et al (2017)^45^	Brazil	Mean (SD) annual cost per patient with MS 0-17 y (USD): 12 295.33 (4001.04)
Lavery et al (2016)^16^	US	Mean, median (SD) cost for an encounter (USD) per patient with MS: 38 543, 24 672 (54 935)
Wright et al (2017)^17^	US	Total costs (USD) among patients with MS <18 y All settings: 1 511 828 Outpatient: 1 256 969 Inpatient: 204 708 Emergency: 50 151 Mean per-patient costs (USD) All settings: 26 523 Outpatient: 22 052 Inpatient: 3591 Emergency: 880 Mean per encounter costs (USD) All settings: 1017 Outpatient: 970 Inpatient: 2924 Emergency: 418
Brenton et al (2019)^22^	US	58% of sample (patients and controls) reported private insurance
Ross et al (2010)^23^	US	Insurance (%) among patients with MS <18 y Private: 67 Medicaid: 26 None: 7

Control group data reported as applicable to outcome of interest.

Six studies^16–20,44^ (from multiple countries) reported on inpatient hospitalizations with varied lengths of follow-up. For example, 1 study reported 0.5 hospital admissions per year among 92 patients in Denmark,^18^ while 2 studies that collected data in the United States reported 1.2^17^ and 6.0^16^ mean hospital admissions per patient over the course of a 10-year study period (using the Pediatric Health Information System database, which includes data from many tertiary-care pediatric hospitals across the United States, and within a hospital system in the state of Utah, respectively). Otherstudies reported on the percentage of patients (out of varying sample sizes) hospitalized in their study cohorts (eg, 16.5% of patients [n = 659] were hospitalized during a 1-year baseline period^19^ and 86.7% of patients <12 years old [n = 15] were hospitalized for an initial relapse^44^). Compared with healthy matched controls, a greater proportion of patients with POMS were hospitalized (19.4% of 36 patients with POMS vs 0% of 43 healthy controls^20^ and 16.5% of 659 patients with POMS vs 2.0% of 3294 healthy controls^19^ in Canada) or admitted at higher rates (rate ratio [RR], 4.27 [2.92-6.25]^18^ among patients in Denmark, odds ratio [OR] 15.2 [12.0-19.1]^19^ among patients in Canada).

Five studies^17–21^ reported other types of utilization, including ambulatory physician visits, hospital visits, and primary care visits. Marrie et al^20^ (using data from Canada) and von Wyl et al^21^ (using data from Switzerland) reported means of 13 (range, 6-27; n = 36) and 7 (range, 4-10; n = 236) physician visits over an unspecified follow-up period, respectively. Wright et al^17^ reported a mean of 22.7 outpatient visits per patient with POMS (n = 57) over a mean follow-up period of 4.49 years in the United States. Boesen et al^18^ reported a mean of 4.6 primary care and 5.9 hospital visits (which included outpatient hospital visits, such as MS clinics, and hospital admissions) per patient (n = 92) per year in Denmark. Compared with age- and sex-matched healthy controls, patients with POMS had higher marginal RRs of primary care visits (1.41 [1.29-1.54]) and hospital visits (10.74 [8.95-12.90]). One study using data from the United States reported on emergency visits and found a mean of 2.1 emergency department visits (range, 0-40; n = 57) per patient over a mean follow-up period of 4.49 years.^17^

Three studies reported on healthcare costs; none compared the costs of patients with POMS with healthy controls.^16,17,45^ Maia Diniz et al^45^ reported that in Brazil, between 2000 and 2015, the mean annual cost per patient was $12 295.33 in 2017 US dollars (USD), or $14 638.05 in 2022 USD.^46^ Wright et al,^17^ using data from 1 statewide hospital system in the United States, reported a mean per-patient cost of $26 523 across all settings, $22 052 for outpatient visits, $3591 for inpatient stays, and $880 for emergency visits (not including provider professional fees) over a mean follow-up period of 4.49 years in 2014 USD, or $32 781, $27 255, $4438, and $1087 in 2022 USD, respectively.^46^ Using the Pediatric Health Information System database, Lavery et al^16^ reported a mean cost of $38 543 per hospital stay in 2015 USD, or $47 679 in 2022 USD.^46^

Two studies using data from the United States identified that 58%^22^ and 67%^23^ of their patients had private insurance, respectively. Ross et al^22^ additionally reported that 26% of patients had Medicaid and 7% had no health insurance coverage.

### Humanistic Burden

Thirty-six studies reported on QOL outcomes in patients with POMS (**Supplemental Table S3**). Studies collected data from Canada (n = 9), the United States (n = 6), or both (n = 3), as well as Italy (n = 7), Germany (n = 4), and the United Kingdom (n = 2). There was 1 study from each of Brazil, France, Netherlands, Norway, and Serbia. Thirteen studies included patients 18 years old and under, 1 study included patients who were slightly older (<19 years old), and 1 study included patients who were slightly younger (<16 years old). The remaining studies either included children and adolescents (eg, 4-18 years old, n = 13) or only adolescents (eg, 12-18 years old, n = 7). One study did not specify the age range of its pediatric population. Twenty-three studies included control groups, most commonly age- and sex-matched healthy controls.

Studies reported data on QOL, including fatigue-related outcomes, using 10 different tools: The Pediatric Quality of Life Measurement Models (PedsQL^TM^ 4.0, PedsQL^TM^ Multidimensional Fatigue Scale), Fatigue Severity Scale, Godin Leisure Time Exercise Questionnaire, Children’s Global Assessment Scale, Multiple Sclerosis Functional Composite, KIDSCREEN-52, Pediatric Functional Assessment of Chronic Illness Therapy–Fatigue, 25-Foot Walk Test, and the Modified Fatigue Impact Scale. The following paragraphs present the findings from the most common QOL and fatigue measurement tools (the PedsQL^TM^ 4.0 and the PedsQL^TM^ Multidimensional Fatigue Scale) to provide an overview on these outcomes. All outcomes from each study are included in **Tables 4, 5, and 6**.

**Table 4.** PedsQL™ Score Study Outcomes

**Author (Year)**	**Country**	**PedsQL Score™**
Marrie et al (2020)^20^	Canada	Mean (SD) psychosocial HRQoL score on PedsQL^TM^ at first measurement (patients with MS <18 y, healthy controls) 76.13 (15.50) vs 79.57 (12.37) 27.8% vs 16.3% with score >1 SD below mean of healthy participants Mean (SD) physical HRQoL score on PedsQL^TM^ at first measurement (patients with MS vs healthy controls) 81.14 (19.49) vs 89.90 (9.59) 30.6% vs 23.3% with score >1 SD below mean of healthy participants 16.7% patients with MS with physical function impaired at first HRQoL measurement
Florea et al (2020)^30^	France	PedsQL^TM^ inventory report <75 (%) among patients with MS ≤18 y Physical: 20 Emotional: 50 Social: 5 School: 50 Global: 40
Storm van’s Gravesande et al (2019)^26^	Germany, Austria	Mean (SD) self-reported HRQoL scale scores (patients with RRMS 12-18 y vs age-matched healthy controls) Physical health: 74.62 (22.1) vs 86.67 (13.64) Emotional functioning: 63.35 (24.89) vs 71.9 (21.21) Social functioning: 88.73 (17.01) vs 91.96 (12.66) School functioning: 58.15 (24.74) vs 71.88 (19.14)
Storm van’s Gravesande et al (2019)^27^	Germany, Austria	Mean (SD) self-reported HRQoL scale scores among patients with RRMS 12-18 y Physical health: 74.62 (22.1) Emotional functioning: 63.35 (24.89) Social functioning: 88.73 (17.01) School functioning: 58.15 (24.74) Total HRQoL scale: 71.81 (18.36)
Ghezzi et al (2017)^29^	Italy	Mean (SD) PedsQL^TM^ summary score at baseline, 52-wk follow-up among patients with RRMS 12-16 y Total scale score: 80.3 (13.5), 80.7 (13.9) Physical health: 81.3 (15.9), 81.9 (15.7) Psychosocial health: 79.7 (13.8), 80.1 (14.9)
Toussaint-Duyster et al (2018)^28^	Netherlands	PedsQL^TM^-HRQoL functioning scores >1 SD below the mean among patients with MS 4-17 y (%) Total: 36 Physical: 45 Emotional: 18 Social: 32 School: 46 Psychosocial: 46
Schwartz et al (2018)^24^	US, Canada	Mean (SD) PedsQL^TM^ among patients with MS 10-18 y Physical functioning: 80.17 (18.50) Emotional functioning: 68.03 (23.05) Social functioning: 83.18 (17.22) School functioning: 63.56 (18.50) Psychosocial health summary score: 71.59 (16.06)
Yeh et al 2017)^25^	US, Canada	Mean PedsQL^TM^ score for patients with MS 10-18 y in interventional control group, motivational interview group Physical functioning: Baseline 82.75, 81.88; 6-mo follow-up 75.13, 83.46 Emotional functioning: Baseline 72.04, 70.20; 6-mo follow-up 65.60, 67.71 Social functioning: Baseline 82.04, 85.20; 6-mo follow-up 79.60, 85.83 School functioning: Baseline 66.11, 66.80; 6-mo follow-up 64.00, 66.88 3-mo follow-up data also included

Abbreviations: HRQoL, health-related quality of life; PedsQL™, Pediatric Quality of Life Measurement Model; RRMS, relapsing-remitting multiple sclerosis.

Control group data reported as applicable to outcome of interest.

**Table 5.** Other QOL Test Study Outcomes

**Author (Year)**	**Country**	**Other QOL Tests**
Fragoso et al (2013)^47^	Brazil	12% of patients with MS <18 y used physiotherapy and hydrotherapy
Grover et al (2016)^41^	Canada	Median (IQR) (patients with MS 12-18 y vs healthy controls) Physical activity self-efficacy: 1.4 (0.6) vs 1.6 (0.4) Perceived disability: 4.0 (7.0) vs 2.0 (3.0) Min/day of total physical activity Median (IQR) measured via accelerometer: 106.3 (60.1) vs 105.0 (68.5) Mean (SD) self-reported measured via GLTEQ: 43.4 (32.6) vs 67.5 (33.2)
Kinnett-Hopkins et al (2016)^42^	Canada	Median (IQR) min/day of total physical activity measured via accelerometer (patients with MS 8-18 y vs healthy controls): 106.33 (60.11), 109.00 (78.55) Median (IQR) self-reported exercise (min/wk) measured via GLTEQ: 36.00 (41.00) vs 65.00 (36.50)
Stephens et al (2019)^48^	Canada	Mean (SD) physical activity level using GLTEQ among patients with MS <18 y Light activity: 9.1 (8.3) Moderate activity: 15.99 (12.2) Vigorous activity: 20.1 (21.5) Health Contribution Score/moderate-to-vigorous physical activity: 35.5 (30.1)
Blaschek et al (2013)^49^	Germany	Mean (SD) sec on timed 25-Foot Walk Test among patients with MS 12-17 y: 3.73 (1.3)
Toussaint-Duyster et al (2018)^28^	Netherlands	Movement Assessment Battery for Children among patients with MS 4-17 y (% total impairment score, manual dexterity, balance) Normal: 48, 62, 52 Borderline: 5, 29, 24 Motor problem: 48, 10, 24
Ostojic et al (2016)^50^	Serbia	Mean (SD) PedsFACIT-F total score among patients with MS 14-18 y: 39.67 (9.32) Mean (SD) KIDSCREEN-52 (patients with MS vs healthy controls) Physical well-being: 47.00 (11.25) vs 52.60 (11.62) Psychological well-being: 49.82 (12.76) vs 51.19 (10.26) Moods and emotions: 51.65 (12.48) vs 47.06 (10.27) Self-perception: 50.74 (10.19) vs 47.83 (8.28) Autonomy: 53.40 (10.88) vs 53.76 (11.01) Parent relation and home life: 55.12 (9.68) vs 50.69 (9.33) Financial resources: 51.96 (8.70) vs 50.43 (8.84) Social support and peers: 52.91 (13.55) vs 54.57 (11.26) School environment: 47.65 (10.24) vs 48.63 (8.97) Social acceptance (bullying): 50.86 (12.67) vs 52.60 (9.13)
Kirk and Hinton (2019)^51^	UK	“The change and changeable body” emerged as the main finding and captured patients’ (8-17 y) experiences of living with an MS diagnosis: altered sense of their identity, changed relationships (particularly with mothers), and a reconfigured future (acknowledging MS would be part of their lives)
Aaen et al (2019)^52^	US	Walked after 15 mo (%) Patients with MS onset <11 y: 3.7 Patients with MS onset ≥11 y: 1.8 Healthy controls: 5.7
Waldman et al (2016)^53^	US	Difference in composite *z* scores on MSFC between patients with MS 6-21 y and healthy controls (OR, *P* value): 0.56, 0.23
Weisbrot et al (2014)^54^	US	Mean (SD) CGAS in patients with MS 8-17 y With psychiatric disorder: 58.20 (15.0) No psychiatric disorder: 88.50 (8.1) (*P *< .001)

Abbreviations: CGAS, Children’s Global Assessment Scale; GLTEQ, Godin Leisure Time Exercise Questionnaire; IQR, interquartile range; MSFC, Multiple Sclerosis Functional Composite; PedsFACIT-F, Pediatric Functional Assessment of Chronic Illness Therapy–Fatigue.

Control group data reported as applicable to outcome of interest.

**Table 6.** Fatigue Study Outcomes

**Author (Year)**	**Country**	**Fatigue**
Akbar et al (2016)^55^	Canada	Mean (SD) PedsQL™ multidimensional fatigue score (patients with MS ≤18 y vs non-MS self-reported healthy individuals): 30.4 (13.3) vs 22.6 (9.01)
Akbar et al (2016)^56^	Canada	Mean (SD) PedsQL™ multidimensional fatigue score (patients with MS <18 y vs healthy controls): 30.8 (14.1) vs 21.9 (7.1)
Akbar et al (2016)^57^	Canada	Mean (SD) PedsQL™ multidimensional fatigue score (patients with MS ≤18 y vs age- and sex-matched healthy controls): 30.0 (13.4) vs 23.3 (8.8)
Fuentes et al (2012)^31^	Canada	23% patients with RRMS <19 y experienced fatigue
Grover et al (2016)^41^	Canada	Median (IQR, % moderate-to-severe) fatigue (Varni Pediatric QOL Inventory Multidimensional Fatigue) (patients with MS vs healthy controls) General: 7.0 (6.0, 15) vs 7.0 (3.0, 14) Cognitive: 7.0 (6.0, 19) vs 7.0 (7.0, 11) Mean (SD, % moderate-to-severe) fatigue Sleep/rest: 7.8 (4.8, 22) vs 9.3 (3.5, 22) Total: 21.3 (12.5; 15) vs 23.1 (8.2, 11)
Stephens et al (2019)^48^	Canada	Baseline mean (SD) fatigue (PedsQL™) among patients with MS <18 y Total: 69.5 (16.5) General: 71.77 (18.9) Sleep/rest: 64.66 (19.88) Cognitive: 72.1 (21.0) Fatigue scores over time estimate (SE) Total: -2.84 (1.03) General: -3.68 (1.18) Sleep/rest: -2.62 (1.05) Cognitive: -1.59 (1.32) *T* values and *P* values also included
Till et al (2012)^39^	Canada	Experienced chronic fatigue: 43.8% patients with RRMS <18 y vs 0% age- and sex-matched healthy controls
Florea et al (2020)^30^	France	43% moderate or severe fatigue on FSS
Storm van’s Gravesande et al (2019)^26^	Germany, Austria	Mean (SD) self-reported fatigue scale scores (PedsQL™) (MS children, age-matched healthy controls) General: 63.15 (25.73), 77.64 (17.83) Sleep/rest: 55.46 (21.69), 63.15 (19.16) Cognitive: 65.99 (26.36), 74.52 (20.76)
Storm van’s Gravesande et al (2019)^27^	Germany, Austria	Mean (SD) self-reported fatigue scale scores (PedsQL™) (patients with RRMS 12-18 y vs age-matched healthy controls) General: 63.15 (25.73) vs 77.64 (17.83) Sleep/rest: 55.46 (21.69) vs 63.15 (19.16) Cognitive: 65.99 (26.36) vs 74.52 (20.76) Total: 61.57 (20.78) vs 71.78 (15.58) Classification of total fatigue (%) (patients with MS vs age-matched healthy controls) None: 59.4 vs 82.8 Mild: 20.8 vs 14.4 Severe: 19.8 vs 2.9 Classification of general, sleep/rest, and cognitive fatigue also included
Kapanci et al (2019)^40^	Germany	Mean (SD) fatigue per Modified Fatigue Impact Scale (patients with MS vs age-and sex-matched healthy controls): 32.52 (17.22) vs 23.10 (12.75)
Amato et al (2014)^58^	Italy	20% of patients with MS <18 y with fatigue on the FSS
Amato et al (2010)^59^	Italy	21% with fatigue on the FSS
De Meo et al (2017)^37^	Italy	Mean (SD) FSS among patients with MS 7-18 y: 27.1 (12.1)
Goretti et al (2012)^35^	Italy	Mean (SD) of self-reported fatigue (PedsQL™) (patients with MS ≤18 y vs demographically matched healthy controls) General: 78.5 (18.9) vs 74.2 (14.1) Sleep: 79.2 (14.3) vs 74.3 (14.5) Cognitive: 83.0 (15.0) vs 77.5 (17.9)
Pastò et al (2016)^60^	Italy	Mean (SD) FSS for patients with RRMS <18 y with cognitive performance that is: Deteriorating: 3.3 (1.9) Stable/improving: 2.5 (1.8)
Rocca et al (2016)^36^	Italy	Mean (SD) FSS All patients with RRMS 8-18 y: 27.2 (12.3) CP patients: 27.6 (11.8) CI patients: 26 (14.2)
Toussaint-Duyster et al (2018)^28^	Netherlands	<1 SD below the mean on PedsQL™ fatigue scores among patients with MS 4-17 y Total: 36 General: 36 Sleep-rest: 27 Cognitive: 32
Sandvig et al (2015)^32^	Norway	61.1% patients with RRMS <16 y reported fatigability
Carroll et al (2016)^61^	UK	Five themes emerged from interviews with patients with MS 6-18 y: (1) emotional reactions to fatigue and its impact, (2) the lived experience of fatigue and impact on daily activities, (3) uncontrollability and unpredictability of fatigue (uncontrollability, uncertainty, and lack of knowledge), (4) finding a balance (concern about well-being, future), (5) social support and disclosure
Parrish et al (2013)^62^	US, Canada	Mean (SD, % moderate-to-severe elevation) fatigue (n = 24) (Varni Pediatric QOL Inventory Multidimensional Fatigue) (patients with MS 10-18 years vs healthy controls) Total: 30.04 (18.48, 29.17) vs 20.03 (10.58, 8.62) General: 9.42 (6.42, 33.33) vs 4.91 (3.68, 3.45) Cognitive: 10.75 (7.26, 41.67) vs 7.05 (5.12, 20.69) Sleep/rest: 9.46 (6.37, 37.5) vs 8.05 (4.57, 20.69)
Charvet et al (2016)^38^	US	Mean (SD, range) FSS among patients with MS 5-18 y: 30.06 (14.37, 9-53) (n = 46)
Holland et al (2014)^63^	US	Mean (SD, range) PedsQL™ Multidimensional Fatigue Scale among patients with MS 7-18 y Cognitive: 61.80 (23.20, 17-106) Sleep/rest: 62.12 (22.50, 17-100) General: 66.88 (19.53, 25-100) Total: 63.08 (18.06, 25-100)
Zafar et al (2012)^64^	US	Mean (SD) total scores (patients with MS 13-18 y vs healthy children in a historical control group) PedsQL™ Multidimensional Fatigue Scale: 61.53 (19.27) vs 61.06 (17.16) Adolescent Sleep-Wake Scale: 4.11 (0.89) vs 5.07 (0.77) Adolescent Sleep Hygiene Scale: 4.48 (0.64) vs 4.35 (0.56) Modified Epworth Sleepiness Scale: 7.00 (3.36) vs 9.44 (4.14)

Abbreviations: CI, cognitively impaired; CP, cognitively preserved; FSS, Fatigue Severity Scale; IQR, interquartile range; PedsQL™, Pediatric Quality of Life; QOL, quality of life; RRMS, relapsing-remitting multiple sclerosis.

Control group data reported as applicable to outcome of interest.

**Quality of life:** Eight studies from various countries used either the PedsQL^TM^ 4.0 or the PedsQL^TM^ Multidimensional Fatigue Scale or both to report patient QOL.^20,24–30^ Overall, higher mean scores (out of 100) indicate better health-related QOL, although no standard categories exist.^33^ One study in our review, from Canada, presented PedsQL^TM^ 4.0 scores of healthy patients^20^; authors reported scores of 89.90 on the physical health component and 79.57 on the psychosocial summary score. Outside the MS literature, 1 study on sickle cell disease pain classified a score of 81 to 100 as “better quality of life” and below 60 as “impaired quality of life.”^34^

Only 2 studies reported total scores. One study from Italy reported a total PedsQL^TM^ 4.0 summary score of 80.3 at baseline and 80.7 at 1-year follow-up.^29^ A study from the Netherlands reported that 36% of patients 4 to 17 years old (n = 22) had abnormal PedsQL^TM^ 4.0 total functioning scores (defined as <1 SD below the mean of healthy age-matched children).^28^

All 8 studies^20,24–30^ reported on the physical health component of the PedsQL^TM^ 4.0, which ranged from 74.62 to 82.75 at baseline; 3 studies^20,24,29^ reported on the psychosocial summary score, which ranged from 71.59 to 79.7 at baseline. One study from France reported that 20% of their patients (n = 26) had scores under 75 on the physical component (considered to be poor QOL).^30^ Another study from the Netherlands reported that 45% and 46% of patients (n = 22) had abnormal physical and psychosocial summary scores, respectively.^28^

**Fatigue**: Fatigue was the most commonly reported QOL outcome among the 24 studies included in our review. Twelve studies used the PedsQL^TM^ Multidimensional Fatigue Scale, which includes a patient self-reported fatigue scale. Mean scores on the self-reported general fatigue scale ranged from 63.15 in German and Austrian patients^26^ to 78.5 in Italian patients^35^ (similar to mean scores in healthy matched controls [74.2-77.64] from the same Italian center). Additionally, compared with age-matched healthy controls, a larger proportion of patients with POMS reported severe fatigue (19.8% vs 2.9%) using patient self-reported data from Germany and Austria.^27^

Seven studies reported on fatigue using the FSS. Mean scores can be calculated 2 ways, and studies in our review reported both. The first method involves averaging numerical responses across all 9 items (ranging from 1 to 7) with higher scores representing greater fatigue severity.^65^ While our review did not include any studies that compared the FSS with healthy controls, the mean score for healthy individuals is 2.3, and a score of 4 or more is considered indicative of problematic fatigue.^66^ One study from Italy included in our review reported a mean FSS on this scale of 2.5 to 3.3.^60^

The second method is based on the sum of each of the 9 items’ individual scores (yielding results ranging from 9 to 63). Although no standard categories exist for this scoring scale, an older study on fatigue in patients with MS (not included) reported the median for healthy controls on the FSS to be 21.5.^67^ Three studies in our review (2 from Italy and 1 from the United States) reported the FSS on this scale, with means ranging from 27.1 to 30.06.^36–38^ Four studies reported on fatigue using the percentage of patients self-reporting fatigue (range, 23%-61.1%)^31,32,39,61^ with 1 study from Canada finding a larger proportion of patients with POMS reporting chronic fatigue (43.8% vs 0.0%) compared with age-matched healthy controls.^39^ One study reported on fatigue using the Modified Fatigue Impact Scale, presenting a mean of 32.52 among patients with POMS compared with 23.10 among age- and sex-matched healthy controls.^40^

**Other QOL outcomes:** Two studies included in our review, both from the United Kingdom, used a qualitative design.^51,61^ Carroll et al^61^ reported on emotional reactions to fatigue, its impact on daily activities, and how patients with POMS (6-18 years old) find balance and social support. Kirk and Hinton^51^ reported on how patients with POMS (8-17 years old) describe that the disease alters their sense of identity, changes relationships, and reshapes how they think about the future. Five studies reported on physical activity outcomes.^41,42,48,49,52^ Two of these studies, both from Canada, compared scores on the Godin Leisure Time Exercise Questionnaire in patients with POMS to healthy matched controls, both reporting fewer exercise minutes per week among patients with POMS (mean, 43.4 vs 67.5^41^; median, 36.00 vs 65.00).^42^

## DISCUSSION

Our review shows that POMS is associated with significant societal and individual burden (both economic and humanistic). Studies reported 1.2 to 6.0 mean hospital admissions per patient over 10 years,^16,17^ high rates of hospitalizations (86%^44^ of the study population), and frequent visits (eg, means of 4.6 primary care and 5.9 hospital visits per year^18^). Patients with POMS had higher rates of primary care visits (RR, 1.41 [1.29- 1.54]), hospital visits (RR, 10.74 [8.95-12.90]), and overall admissions (RR, 4.27 [2.92-6.25] and OR 15.2 [12.0-19.1]) compared with healthy controls.^18,19^ Mean per-patient costs were $5907 across all settings per year of follow-up between 2002 and 2012,^17^ and mean costs per hospital stay were $38 543 between 2004 and 2013.^16^

Given that incidence, prevalence, and costs of POMS vary by geography (eg, lower per-patient costs were reported in Brazil^45^ compared with the United States^16,17^), it is difficult to generalize the global economic burden. We did not identify any published reviews on healthcare resource utilization or costs in patients with POMS. The US total cost of $5907 ($7524 in 2022 USD)^46^ per patient per year of follow-up is a low estimate because this calculation excluded provider professional fees. Furthermore, these costs did not account for insurance coverage. However, this cost is still higher than overall costs in the US pediatric population, which has been reported to be less than $3000 ($3755 in 2022 USD)^46^ per child across all settings and age groups.^68^ Reviews based on adult patients with MS reported higher costs in the United States than our pediatric findings (total mean all-cause healthcare costs for adults ranged from $8528 to $54 244 per patient per year in 2011^14^) and lower costs in Spain (mean annual cost per patient of €30 050,^13^ equivalent to $43 259 in 2011 USD or $56 790 in 2022 USD).^69^

Even with treatment, patients with POMS have reduced QOL compared with healthy controls. Studies reported PedsQL^TM^ 4.0 psychosocial scores between 72 to 80^20,24,29^ and physical health scores between 75 to 83.^20,24–30^ Studies of children with cancer reported similarly low mean psychosocial scores (73) and even lower mean physical health scores (72).^70^ Compared with healthy controls reported in our review, both patients with POMS and those with cancer have lower scores than healthy controls (mean psychosocial scores between 79 to 89^20^ and physical health scores between 82 to 84^70^). Patients also reported problematic fatigue (FSS scores of patients in studies included in our review ranged from approximately 27 to 30, compared with a median score of 21 for healthy controls in a 2003 study of MS patients^67^). In adults with MS, symptoms of fatigue have been shown to impact disease course and functional outcomes.^71^ In children and adolescents already experiencing the typical challenges of growing up, reduced QOL, including impaired mental, physical, emotional, and academic functioning, may have a greater impact on life trajectories than in adults with MS.^8^

Our review of QOL studies found similar articles to Carroll et al,^10^ a recent systematic review on fatigue and its association with clinical, psychological, and social factors in children and adolescents with POMS. Six of the 12 studies included in the prior review were identified in ours. Those which were not included were either published prior to our search timeline (4 studies) or focused on cognitive tests or behavioral health outcomes (2 studies) that we did not consider to be aligned with our QOL outcomes. As a result, their study concluded similar findings to ours, namely that fatigue is a concerning and common symptom in children and adolescents with POMS.

This review has limitations. While our goal was to report on patients 10 to 17 years old (other studies included wider age ranges), we included all studies that presented data on the pediatric population. Our results are not specific to adolescents and include data from some very young patients with POMS. Second, while studies reported on different types of MS (eg, relapsing remitting vs primary progressive), we did not examine nor report on these differences. Lastly, we did not assess the quality of the studies included in our review or whether any author bias was present.

Our search identified gaps in the current literature and yielded ideas for future research. We found fewer studies in our review that reported on healthcare resource utilization and costs than on QOL outcomes. POMS is costly, and additional studies, such as studies utilizing healthcare claims, should further estimate the costs associated with POMS. Further, studies that consistently report costs over the same time frame (eg, per year) would help compare findings across publications. Only 2 studies reported on patient insurance coverage. Studies exploring patient insurance coverage and potential lack of coverage would provide a more complete picture of the economic burden these young patients and their families face. Furthermore, given the reduced QOL in these patients, clinical trials should continue to measure QOL and fatigue, documenting how therapies may help address these outcomes. Lastly, while several studies reported on PedsQL^TM^ and FSS measures, the QOL studies reported on a wide range of instruments. More consistent reporting of a limited number of instruments would have made comparing results more informative.

## CONCLUSION

Our review presents a uniquely broad and recent overview of the global economic and humanistic burden of patients with POMS. While no articles presented both costs and QOL, our review demonstrates that healthcare resource utilization and costs are high in this population, and patients report reduced QOL and significant fatigue compared with healthy children and adolescents. This decrease in QOL and increase in economic burden in children and adolescents may have further reaching consequences than those in older patients. Children this age are already experiencing challenges typical of this difficult development stage, burden is more likely to affect the entire family rather than the patient alone, and consequences may impact the child’s potential for future achievements.

### Author Contributions

All authors were involved in design, data acquisitions, and/or data analysis and interpretation. All authors were involved in development of the manuscript and providing critical review. All authors approved the final draft and take responsibility for the content.

### Disclosures

N.G. and L.A. are full-time employees of Sanofi and may hold stock and/or stock options. C.C., H.D., S.N.G. and I.Y. are employees of PHAR (Partnership for Health Analytic Research), a health services research company paid by Sanofi to conduct the literature review.

## Supplementary Material

Online Supplementary Material
