# Supplementary material for: The Economic and Humanistic Burden of Pediatric-Onset Multiple Sclerosis
Source: J Health Econ Outcomes Res. 2022 Oct 18;9(2):103–14. doi: 10.36469/001c.37992 (PMC9584745; doi:10.36469/001c.37992)
Supplement: Online Supplementary Material [file jheor_2022_9_2_37992_102137.pdf]

Journal of Health Economics and Outcomes Research

Online Supplementary Material

The Economic and Humanistic Burden of Pediatric-Onset Multiple Sclerosis. *JHEOR*. 2022;9(2):103-114. doi:10.36469/jheor.2022.37992

Table S1: Search Strategy

Table S2: Healthcare Resource Utilization, Healthcare Cost, and Health Insurance Coverage Studies Included

Table S3: Quality-of-Life Studies Included

This supplementary material has been provided by the authors to give readers additional information about their work.

This is an open-access article distributed under the terms of the Creative Commons Attribution 4.0 International License (CCBY-4.0). View this license's legal deed at http://creativecommons.org/licenses/by/4.0 and legal code at http://creativecommons.org/licenses/by/4.0/legalcode for more information.

Table S1. Search Strategy

Topic of Interest	PubMed Search String	Embase Search String
Pediatric-onset multiple sclerosis	(“Pediatric onset multiple sclerosis”[tw] OR “POMS”[tiab] OR “pediatric multiple sclerosis”[tw] OR ‘multiple sclerosis’[tw] OR ‘multiple sclerosis’[Mesh]) AND	((‘pediatric onset multiple sclerosis’/exp OR ‘POMS’:ti,ab,kw OR ‘pediatric multiple sclerosis’:ti,ab,kw OR ‘multiple sclerosis’/exp OR ‘multiple sclerosis’:ti,ab,kw) AND
Children, adolescents	(“Child”[Mesh] OR “Adolescent”[Mesh] OR child*[tw] OR kid[tw] OR kids[tw] OR teen*[tw] OR adolescen*[tw] OR youth*[tw] OR pediatric*[tw] OR “Pediatrics”[Mesh]) AND	(‘child’/exp OR ‘adolescent’/exp OR child*:ti,ab,kw OR kid*:ti,ab,kw OR teen*:ti,ab,kw OR adolescen*:ti,ab,kw OR youth*:ti,ab,kw OR pediatric*:ti,ab,kw OR ‘pediatrics’/exp) AND
Humanistic disease burden, morbidity, comorbidities, health-related quality of life	“disease burden*”[tw] OR “burden of disease*”[tw] OR morbidit*[tw] OR “Morbidity”[Mesh] OR “Comorbidity”[Mesh] OR “comorbidit*”[tw] OR “co-morbidit*”[tw] OR “illness*”[tw] OR “disease”[Mesh] OR “disease*”[tw] OR “burden of illness”[tw] OR “illness burden*”[tw] OR “Quality of Life”[Mesh] OR “quality of life”[tw] OR “HRQOL”[tw] OR “health related quality of life”[tw] OR	‘disease burden’/exp OR ‘disease burden’:ti,ab,kw OR ‘burden of disease’:ti,ab,kw OR morbidit*:ti,ab,kw OR ‘morbidity’/exp OR ‘comorbidity’/exp OR ‘comorbidit*’:ti,ab,kw OR ‘co-morbidit*’:ti,ab,kw OR ‘diseases’/exp OR disease*:ti,ab,kw OR illness*:ti,ab,kw OR ‘burden of illness’/exp OR ‘burden of illness’:ti,ab,kw OR ‘illness burden*’:ti,ab,kw OR ‘quality of life’/exp OR ‘quality of life’:ti,ab,kw OR ‘HRQOL’:ti,ab,kw OR ‘health related quality of life’:ti,ab,kw OR
Economic disease burden, healthcare utilization, healthcare costs, health insurance reimbursement	“Cost of Illness”[Mesh] OR “economic disease burden”[tw] OR “economic burden of disease”[tw] OR “Patient Acceptance of Health Care”[Mesh] OR “healthcare utilization”[tw] OR “health care utilization”[tw] OR “Health Care Costs”[Mesh] OR “health care cost*”[tw] OR “healthcare cost*”[tw] OR “medical care cost*”[tw] OR “medical cost*”[tw] OR “Insurance, Health, Reimbursement”[Mesh] OR reimbursement*[tw] OR “Nursing Homes”[Mesh] OR “nursing home*”[tw] OR “care facilit*”[tw] OR “Long-Term Care”[Mesh] OR “long term care”[tw] OR “formal care”[tw] OR “informal care”[tw] OR “productivity loss”[tw] OR “loss of productivity”[tw] OR “Health Resources”[Mesh] OR “health resource*”[tw])	‘cost of illness’/exp OR ‘economic disease burden’:ti,ab,kw OR ‘economic burden of disease’:ti,ab,kw OR ‘health care utilization’/exp OR ‘healthcare utilization’:ti,ab,kw OR ‘health care utilization’:ti,ab,kw OR ‘health care cost’/exp OR ‘health care cost*’:ti,ab,kw OR ‘healthcare cost*’:ti,ab,kw OR ‘medical care cost*’:ti,ab,kw OR ‘medical cost’/exp OR ‘medical cost*’:ti,ab,kw OR ‘nursing home’/exp OR ‘nursing home*’:ti,ab,kw OR ‘care facility*’:ti,ab,kw OR ‘long term care’/exp OR ‘long term care*’:ti,ab,kw OR ‘formal care’/exp OR ‘formal care’:ti,ab,kw OR ‘informal care’:ti,ab,kw OR ‘informal care’/exp OR ‘productivity loss’/exp OR ‘productivity loss’:ti,ab,kw OR ‘loss of productivity’:ti,ab,kw OR ‘health resource*’:ti,ab,kw OR ‘reimbursement’/exp OR ‘reimbursement’:ti,ab,kw))
Other	NOT (Case Reports[ptyp] OR “in vitro”)<br>Sort by “Most recent”<br>Add “English” and “Last 10 years” filters	NOT (‘case report’/exp OR ‘in vitro study’/exp)<br>Exclude conference abstracts, editorials, conference reviews, letters, notes, conference papers, chapters; include articles, reviews, articles in press, short surveys, erratum<br>Add “English” and “Last 10 years” filters

Table S2. Healthcare Resource Utilization, Healthcare Cost, and Health Insurance Coverage Studies Included

Author (Year)	Title	Study Design	Patient Population	Control Group	Data Source	Country	Data Period
Maia Diniz et al (2017)[23]	The Long-term Costs for Treating Multiple Sclerosis in a 16-Year Retrospective Cohort Study in Brazil	Registry	Patients with MS 0-65+ y who started a DMT (N = 23082), stratified by patients 0-17 y (N = 833)	None	Patient-centered registry obtained through the Outpatient Information System, Hospital Information System, and Mortality Information Systems in the Brazilian public health system	Brazil	2000-2015
Marrie et al (2020)[18]	Factors Associated With Health Care Utilization in Pediatric Multiple Sclerosis	Healthcare claims analysis and retrospective cohort study	Patients with MS, ADS, and healthy controls <18 y (N = 115), stratified by patients with MS (N = 36)	Healthy controls (N = 43)	Population-based health services data held at ICES	Canada	2004-2018
Marrie et al (2019)[19]	High Rates of Health Care Utilization in Pediatric Multiple Sclerosis: A Canadian Population-Based Study	Healthcare claims analysis	Patients with MS ≤18 y (N = 659)	Age-, sex-, and region-matched healthy controls (N = 3294)	Population-based health services data held at ICES	Canada	2003-2014
Boesen et al (2020)[16]	School Performance, Psychiatric Comorbidity, and Healthcare Utilization in Pediatric Multiple Sclerosis: A Nationwide Population-Based Observational Study	Retrospective cohort study and registry	Patients with MS <18 y (N = 92)	Age- and sex-matched healthy controls (N = 920) and patients with non–brain-related chronic disease (N = 9108)	Hospital records and Danish Multiple Sclerosis Registry	Denmark	2008-2015
Boesen et al (2019)[59]	Infections Seem to Be More Frequent Before Onset of Pediatric Multiple Sclerosis: A Danish Nationwide Nested Case-Control Study	Nested case-control study	Patients with MS <18 y (N = 212)	Age- and sex-matched controls (N = 1060)	Danish Multiple Sclerosis Registry	Denmark	1995-2015
von Wyl et al (2020)[22]	Influence of Age at Disease Onset on Future Relapses and Disability Progression In Patients With Multiple Sclerosis on Immunomodulatory Treatment	Healthcare claims analysis	Patients with MS any age on DMT for ≥1 year (N = 9705), stratified by patients <18 y (N = 236); RRMS=217, CIS=19	None	Swiss Association for Joint Tasks of Health Insurers database	Switzerland	1995-2017
Brenton et al (2019)[25]	Body Mass Index Trajectories in Pediatric Multiple Sclerosis	Case-control study	Patients with MS <18 y (N = 40)	Age-, sex-, ethnicity-, and socioeconomic status-matched typically developing controls (N = 120)	Pediatric Multiple Sclerosis and Related Disorders Clinic, University of Virginia	US	2016-2017

Table S2. Healthcare Resource Utilization, Healthcare Cost, and Health Insurance Coverage Studies Included, *cont'd*

Author (Year)	Title	Study Design	Patient Population	Control Group	Data Source	Country	Data Period
Lavery et al (2016)[17]	Hospital Admission Rates for Pediatric Multiple Sclerosis in the United States Using the Pediatric Health Information System (PHIS)	Healthcare claims analysis and retrospective cohort study	Patients with MS <19 y (N = 1124)	None	PHIS database, medical records (2004-2013) from Children's Hospital of Philadelphia (used to confirm hospitalization rates)	US	2004-2014
Ross et al (2010)[26]	Neurocognitive Sequelae in African American and Caucasian Children With Multiple Sclerosis	Cross-sectional study	Patients with MS onset <18 (N = 42), stratified by African American (N = 20) and White patients (N = 22)	None	Center for Pediatric-Onset Demyelinating Disorders clinic, University of Alabama, Birmingham	US	2006-2009
Wright et al (2017)[20]	Comprehensive Population-Based Determination of Pediatric Multiple Sclerosis Health Care Costs	Healthcare claims analysis	Patients with MS <18 y (N = 57)	None	Intermountain Healthcare Enterprise Data Warehouse	US	2002-2012
Krupp (2016)[21]	Subcutaneous Interferon β-1a in Pediatric Patients With Multiple Sclerosis: Regional Differences in Clinical Features, Disease Management, and Treatment Outcomes in an International Retrospective Study	Retrospective chart review	Patients with MS 0-17 y (N = 298), stratified into preadolescents (<12 y) and adolescents (12-17 y) and by US and ROW	None	Medical records	US, Italy, Russia, Argentina, France, Canada, Tunisia, Venezuela	1997-2009

Abbreviations: ADS, acquired demyelinating syndromes; CIS, clinically isolated syndrome; DMT, disease-modifying therapy; ICES, Institute for Clinical Evaluative Sciences; RRMS, relapsing-remitting multiple sclerosis; ROW, rest of world.
Outcome data listed in **Tables 2 and 3.**

Table S3. Quality-of-Life Studies Included

Author (Year)	Title	Study Design	Patient Population	Control Group	Data Source	Country	Data Period
Fragoso et al (2013)[60]	Multiple Sclerosis Starting Before the Age of 18 Years: The Brazilian Experience	Retrospective chart review	Patients with MS <18 y (N = 125)	None	20 MS clinics throughout Brazil	Brazil	2012
Akbar et al (2016)[65]	Altered Resting-State Functional Connectivity in Cognitively Preserved Pediatric-Onset MS Patients and Relationship to Structural Damage and Cognitive Performance	Cross-sectional study	Patients with MS <18 y (N = 16)	Age- and sex-matched healthy controls (n = 15)	MS programs at 2 Toronto hospitals	Canada	2016 (publication date)
Akbar et al (2016)[66]	Alterations in Functional and Structural ConnectivityiIn Pediatric-Onset Multiple Sclerosis	Cross-sectional study	Patients with MS <18 y (N = 19)	Age- and sex-matched healthy controls (n = 16)	Hospital for Sick Children	Canada	2016 (publication date)
Akbar et al (2016)[64]	Brain Activation Patterns and Cognitive Processing Speed in Patients With Pediatric-Onset Multiple Sclerosis	Cross-sectional study	Patients with MS ≤18 y (N = 20)	Non-MS self-reported healthy individuals (n = 16)	MS programs at 2 Toronto hospitals	Canada	2016 (publication date)
Fuentes et al (2012)[45]	Memory Performance and Normalized Regional Brain Volumes in Patients With Pediatric-Onset Multiple Sclerosis	Retrospective chart review	Patients with RRMS <19 y (N = 32)	Age- and sex-matched healthy controls (n = 26)	Participants from serial study at the Pediatric MS Clinic, Hospital for Sick Children	Canada	2007-2010
Grover et al (2016)[50]	Physical Activity and Its Correlates in Youth With Multiple Sclerosis	Cross-sectional study	Patients with ADS or MS 12-18 y (N = 68), stratified by patients with MS (n = 27)	Healthy controls (n = 37)	Multiple Sclerosis and Neuroinflammatory Clinic, Hospital for Sick Children	Canada	2016 (publication date)
Kinnett-Hopkins et al (2016)[53]	Physical Activity in Pediatric Onset Multiple Sclerosis: Validating a Questionnaire for Clinical Practice and Research	Cross-sectional study	Patients with MS 8-18 y (N = 27)	Healthy controls (n = 45)	Pediatric MS and Neuroinflammatory Clinic, Hospital for Sick Children	Canada	2016 (publication date)
Marrie et al (2020)[18]	Factors Associated With Health Care Utilization in Pediatric Multiple Sclerosis	Healthcare claims analysis and retrospective cohort study	Patients with MS, ADS, and healthy controls <18 y (N = 115), stratified by patients with MS (n = 36)	Healthy controls (n = 43)	Population-based health services data held at ICES	Canada	2004-2018
Stephens et al (2019)[54]	Benefits of Physical Activity for Depression and Fatigue in Multiple Sclerosis: A Longitudinal Study	Prospective cohort study	Patients with MS <18 y (N = 182), stratified by patients with mono-ADS (n = 134) or MS (n = 48)	None	Pediatric Neuroinflammatory Disorders Clinic, Hospital for Sick Children	Canada	2013-2017
Till et al (2012)[47]	Factors Associated With Emotional and Behavioral Outcomes in Adolescents With Multiple Sclerosis	Case-control study	Patients with RRMS onset <18 y and 12-19 y at time of assessment (N = 31)	Age- and sex-matched healthy controls (n = 31)	Pediatric Demyelinating Disease Clinic, Hospital for Sick Children	Canada	2012 (publication date)

Table S3. Quality-of-Life Studies Included, *cont'd*

Author (Year)	Title	Study Design	Patient Population	Control Group	Data Source	Country	Data Period
Florea et al (2020)[30]	Fatigue, Depression, and Quality of Life in Children With Multiple Sclerosis: A Comparative Study With Other Demyelinating Diseases	Prospective cohort study	Patients with MS or ADS (N = 37), stratified by patients with MS ≤18 y (n = 26)	None	National Referral Centre for rare brain and spinal diseases of the University Hospital of Paris-Sud, Bicentre Hospital	France	2014-2016
Storm van's Gravesande et al (2019)[32]	The Multiple Sclerosis Inventory of Cognition For Adolescents (MUSICADO): A Brief Screening Instrument to Assess Cognitive Dysfunction, Fatigue and Loss of Health-Related Quality of Life in Pediatric-Onset Multiple Sclerosis	Cross-sectional study	Patients with RRMS 12-18 y (N = 106)	Age-matched healthy controls (n = 210)	20 German and 2 Austrian Neuropediatric Departments of University Hospitals, 6 German centers	Germany, Austria	2012-2015
Storm van's Gravesande et al (2019)[33]	Fatigue and Depression Predict Health-Related Quality of Life in Patients With Pediatric-Onset Multiple Sclerosis	Cross-sectional study	Patients with RRMS 12-18 y (N = 106)	Age-matched healthy controls (n = 210)	20 German and 2 Austrian Neuropediatric Departments of University Hospitals, 6 German centers	Germany, Austria	2012-2015
Blaschek et al (2013)[52]	Early White Matter Changes in Early Childhood Multiple Sclerosis: A Diffusion Tensor Imaging Study	Prospective cohort study	Patients with MS 12-17 y (N = 14)	Age- and sex-matched healthy controls (n = 14)	Dr. Von Hauner Children's Hospital	Germany	2013 (publication date)
Kapanci et al (2019)[48]	Evaluating the Relationship Between Psychometric Intelligence and Cognitive Functions in Paediatric Multiple Sclerosis	Prospective cohort study	Patients with MS (N = 21); specific age not specified, mean (SD) 15.5 (1.8) y	Age- and sex-matched healthy controls	Hospitals in Germany	Germany	2019 (publication date)
Amato et al (2010)[68]	Cognitive and Psychosocial Features in Childhood and Juvenile MS	Prospective cohort study	Patients with MS onset <18 y (N = 56)	Demographically-matched healthy controls (n = 50)	11 Italian MS centers	Italy	2006-2010 (publication date)
Amato et al (2014)[67]	Neuropsychological Features in Childhood and Juvenile Multiple Sclerosis: Five-Year Follow-Up.	Prospective cohort study	Patients with MS onset <18 y (N = 48)	Demographically-matched healthy controls (n = 46)	11 Italian MS centers	Italy	2006-2014 (publication date)
De Meo et al (2017)[42]	MRI Substrates of Sustained Attention System and Cognitive Impairment in Pediatric MS Patients	Prospective cohort study	Patients with RRMS 7-18 y (N = 57), data stratified by cognitively preserved, cognitively impaired patients, and healthy controls	Age- and sex-matched healthy controls (n = 14)	Specialized MS centers in Italy	Italy	2017 (publication date)

Table S3. Quality-of-Life Studies Included, *cont'd*

Author (Year)	Title	Study Design	Patient Population	Control Group	Data Source	Country	Data Period
Ghezzi et al (2017)[27]	A Multicenter, Observational, Prospective Study of Self- and Parent-Reported Quality of Life in Adolescent Multiple Sclerosis Patients Self-Administering Interferon-β1a Using Rebismart™—The FUTURE Study	Prospective cohort study	Patients with RRMS 12-16 y (N = 50)	None	14 Italian pediatric treatment centers	Italy	2012-2014
Goretti et al (2012)[36]	Fatigue and Its Relationships With Cognitive Function and Depression in Paediatric Multiple Sclerosis	Prospective cohort study	Patients with MS ≤18 y (N = 57)	Demographically matched healthy controls (n = 70)	11 Italian MS centers	Italy	2012 (publication date)
Pastò et al (2016)[39]	The Cognitive Reserve Theory in the Setting of Pediatric-Onset Multiple Sclerosis	Longitudinal cohort study	Patients with RRMS <18 y (N = 48), stratified by CP (n = 34) and CI (n = 14) patients	Age- and sex-matched healthy controls (n = 57)	11 MS Centers	Italy	2006-2007, followed for 5 years
Rocca et al (2016)[41]	Regional Hippocampal Involvement and Cognitive Impairment in Pediatric Multiple Sclerosis	Case-control study	Patients with RRMS 8-18 y (N = 53), stratified by CP (n = 41) and CI patients (n = 12)	Age- and sex-matched healthy controls (n = 18)	Specialized MS centers	Italy	2016 (publication date)
Toussaint-Duyster et al (2018)[3]	Fatigue and Physical Functioning in Children With Multiple Sclerosis and Acute Disseminated Encephalomyelitis	Cross-sectional study	Patients with MS (n = 22) and post-ADEM (n = 16) 4-17 y	None	National multidisciplinary pediatric MS Center	Netherlands	2013-2015
Sandvig et al (2015)[46]	Multiple Sclerosis in Children and Adolescents. An Important Differential Diagnosis of Acute Neurological Disease	Retrospective chart review	Patients with RRMS between <16 y (N = 18)	None	Department of Child Neurology, Oslo University Hospital	Norway	2004-2012
Ostojic et al (2016)[61]	Quality of Life and Its Correlates in Adolescent Multiple Sclerosis Patients	Cross-sectional study	Patients with MS 14-18 y (N = 21)	Healthy controls (n = 110)	2 pediatric university clinics in Belgrade: The Clinic of Neurology and Psychiatry for Children and Youth and The Institute of Mother and Child Healthcare of Serbia, National Data for Healthy Adolescents	Serbia	2016 (publication date)
Carroll et al (2016)[44]	"It Feels Like Wearing a Giant Sandbag." Adolescent and Parent Perceptions of Fatigue in Paediatric Multiple Sclerosis	Qualitative cross-sectional study	Patients with MS 6-18 y (N = 15)	None	NHS specialist pediatric neurology clinics, MS charities, online support forums	United Kingdom	2016 (publication date)
Kirk et al (2019)[49]	"I'm Not What I Used To Be": A Qualitative Study Exploring How Young People Experience Being Diagnosed With a Chronic Illness	Qualitative cross-sectional study	Patients with MS 8-17 y (N = 21)	None	16 specialist pediatric MS centers	United Kingdom	2019 (publication date)

Parrish et al (2013)[69]	Fatigue and Depression in Children With Demyelinating Disorders	Cross-sectional study	Patients with demyelinating disorders 10-18 y (N = 49), stratified by patients with MS (n = 36)	Healthy controls (n = 92)	Pediatric MS Center of Excellence	United States, Canada	2007-2011
Schwartz et al (2018)[28]	Risk Factors for Non-Adherence to Disease-Modifying Therapy in Pediatric Multiple Sclerosis	Prospective cohort study	Patients with MS 10-18 y receiving oral or injectable DMT for at least 6 mo (N = 66)	None	12 pediatric MS clinics including specifically pharmacy-refill data	US, Canada	2013-2016
Yeh et al (2017)[29]	Impact of Electronic Monitoring Device and Behavioral Feedback on Adherence to Multiple Sclerosis Therapies in Youth: Results of a Randomized Trial	Randomized controlled trial	Patients with MS 10-18 y receiving an oral or injectable DMT for at least 6 mo (N = 52), randomized to receive either a motivational interview (behavioral feedback + electronic monitoring device, n = 25) or an MS medication video (attention control group, n = 27)	None	9 pediatric MS clinics	U,s Canada	2013-2016
Aaen et al (2019)[51]	Acquisition of Early Developmental Milestones and Need for Special Education Services in Pediatric Multiple Sclerosis	Case-control study	Patients with MS ≤18 y (N = 467)	Healthy controls (n = 428)	16 MS centers, most of which participate in United States Network of Pediatric Multiple Sclerosis Centers	US	2018 (publication date)
Charvet et al (2016)[43]	Behavioral Symptoms in Pediatric Multiple Sclerosis: Relation to Fatigue and Cognitive Impairment	Prospective cohort study	Patients with RRMS 5-18 y (N = 108)	None	Lourie Center for Pediatric MS of Stony Brook Medicine	US	2006-2014
Holland et al (2014)[70]	Fatigue, Emotional Functioning, and Executive Dysfunction in Pediatric Multiple Sclerosis	Retrospective chart review	Patients with MS 7-18 y (N = 26)	None	Children's Medical Center Dallas Pediatric Demyelinating Diseases Clinic	US	2014 (publication date)
Waldman et al (2016)[62]	Binocular Low-Contrast Letter Acuity and the Symbol Digit Modalities Test Improve the Ability of the Multiple Sclerosis Functional Composite to Predict Disease in Pediatric Multiple Sclerosis	Prospective case-control study	Patients with MS 6-21 y (N = 20)	Age-matched healthy controls (n = 13) required to have normal corrected visual acuity	Pediatric MS Program, Children's Hospital of Philadelphia	US	2007-2012

Weisbrot et al (2014)[63]	Psychiatric Diagnosis and Cognitive Impairment in Pediatric Multiple Sclerosis	Prospective cohort study	Patients with MS 8-17 y (N = 45)	None	Lourie Center for Pediatric MS of Stony Brook Medicine	US	2006-2011
Zafar et al (2012)[71]	Examining Sleep, Fatigue, and Daytime Sleepiness in Pediatric Multiple Sclerosis Patients	Prospective case-control study	Patients with MS 13-18 y (N = 30)	Healthy children in a historical control group (n = 52)	University of Alabama at Birmingham Center for Pediatric Onset Demyelinating Disease, Children's Hospital of Alabama clinic database	US	2008-2009

Abbreviations: ADE, acute disseminated encephalomyelitis; ADS, acquired demyelinating syndromes; CI, cognitively impaired; CP, cognitively preserved; DMT, disease-modifying therapy; ICES, Institute for Clinical Evaluative Sciences; MS, multiple sclerosis; NHS, National Health Service; RRMS, relapsing-remitting multiple sclerosis.
Outcome data listed in **Tables 4, 5, and 6**.
